# Suppression of Radiation-Induced Salivary Gland Dysfunction by IGF-1

**DOI:** 10.1371/journal.pone.0004663

**Published:** 2009-03-02

**Authors:** Kirsten H. Limesand, Sherif Said, Steven M. Anderson

**Affiliations:** 1 Department of Pathology, University of Colorado School of Medicine, University of Colorado Denver, Aurora, Colorado, United States of America; Ordway Research Institute, United States of America

## Abstract

**Background:**

Radiation is a primary or secondary therapeutic modality for treatment of head and neck cancer. A common side effect of irradiation to the neck and neck region is xerostomia caused by salivary gland dysfunction. Approximately 40,000 new cases of xerostomia result from radiation treatment in the United States each year. The ensuing salivary gland hypofunction results in significant morbidity and diminishes the effectiveness of anti-cancer therapies as well as the quality of life for these patients. Previous studies in a rat model have shown no correlation between induction of apoptosis in the salivary gland and either the immediate or chronic decrease in salivary function following γ-radiation treatment.

**Methodology/Principal Finding:**

A significant level of apoptosis can be detected in the salivary glands of FVB mice following γ-radiation treatment of the head and neck and this apoptosis is suppressed in transgenic mice expressing an activated mutant of Akt (myr-Akt1). Importantly, this suppression of apoptosis in myr-Akt1 mice preserves salivary function, as measured by saliva output, three and thirty days after γ-radiation treatment. In order to translate these studies into a preclinal model we found that intravenous injection of IGF1 stimulated activation of endogenous Akt in the salivary glands *in vivo*. A single injection of IGF1 prior to exposure to γ-radiation diminishes salivary acinar cell apoptosis and completely preserves salivary gland function three and thirty days following irradiation.

**Conclusions/Significance:**

These studies suggest that apoptosis of salivary acinar cells underlies salivary gland hypofunction occurring secondary to radiation of the head and neck region. Targeted delivery of IGF1 to the salivary gland of patients receiving head and neck irradiation may be useful in reducing or eliminating xerostomia and restoring quality of life to these patients.

## Introduction

The effects of γ-radiation upon the salivary gland have been known for over ninety years having been first described in the human in 1911 [1]. Radiation-induced damage to the salivary glands results in xerostomia (dry mouth), and affected patients have both compromised quantity and quality of saliva that can affect the emotional and systemic well-being of these individuals [2]. Such patients suffer from increased oral infections, difficulty in speaking, difficulty in swallowing food, and problems with digestion. In the human, the parotid gland is more radiosensitive when compared to the submandibular gland [3], [4]. Various studies have noted over a 50% reduction in parotid gland function within a few days after exposure of the head and neck region to low doses of irradiation [reviewed in [5]]. The high sensitivity of the parotid gland has been suggested to be due to the predominance of serous cells in this gland as these epithelial cells appear to be the most radiosensitive [3], [4].

While numerous experimental models have been used to study the effects of irradiation upon the salivary gland, the rat is the most extensively studied. Prior studies have suggested that damage to the salivary gland occurs in multiple phases [6], [7]. The acute phases (0–60 days) are characterized by a significant decline in salivary flow rate to approximately 50% of control, loss of glandular wet weight and acinar cells, and alterations in water and protein composition of saliva. In the chronic phases (60–240 days), cell number and protein secretion remain unchanged from the acute phase, while salivary flow are approximately 30% of control values [reviewed in [5]].

While there is an appreciation for the changes that are induced on the cellular and physiological level, the molecular mechanism underlying these changes are not understood, and most investigators have suggested that necrosis is responsible for salivary gland hypofunction. Apoptosis has been discounted as causative because a single study in rats has shown that less than 2% of salivary acinar cells are apoptotic six hours following irradiation of rat salivary glands with X-rays with doses as high as 25 Gy [8]. These results seemed at variance with the observed 50% decrease in salivary flow rate detected by three days after x-irradiation at doses of 15 Gy [8], and thus these investigators concluded that radiation-induced apoptosis was not causally related to salivary gland dysfunction.

While there are many molecules that are able to suppress apoptosis, we have focused upon the anti-apoptotic protein kinase Akt which is able to suppress apoptosis in a number of cell systems [9]–[11]. Our previous studies have demonstrated that salivary acinar cells are sensitive to γ-radiation-induced apoptosis in primary cultures *in vitro* and *in vivo* [12]–[14]. We have demonstrated that activated mutants of Akt1 can suppress apoptosis *in vitro* in primary cultures as well as established salivary acinar cell lines [12], [15]. Likewise activation of Akt, mediated by stimulation of cells with IGF1 and EGF can suppress apoptosis *in vitro* [12]. Expression of constitutively activated Akt1 in transgenic mice (myr-Akt1) can reduce apoptosis by 70% following targeted head and neck γ-radiation suggesting that apoptosis may have a larger role in γ-radiation-induced salivary gland dysfunction than originally reported [14]. We report in this study that reduction of γ-radiation-induced apoptosis correlates with improved salivary flow rates in mice. Importantly, a single injection of recombinant IGF1, which stimulates activation of endogenous Akt, completely prevents loss of saliva production 30 days post-irradiation. This suggests a model for the development of a clinical approach to suppress salivary gland dysfunction and thus improve the quality of life in patients undergoing therapeutic head and neck radiation.

## Results

### Reduced apoptosis in myr-Akt1 parotid glands following exposure to ionizing radiation

**Figure 1:**
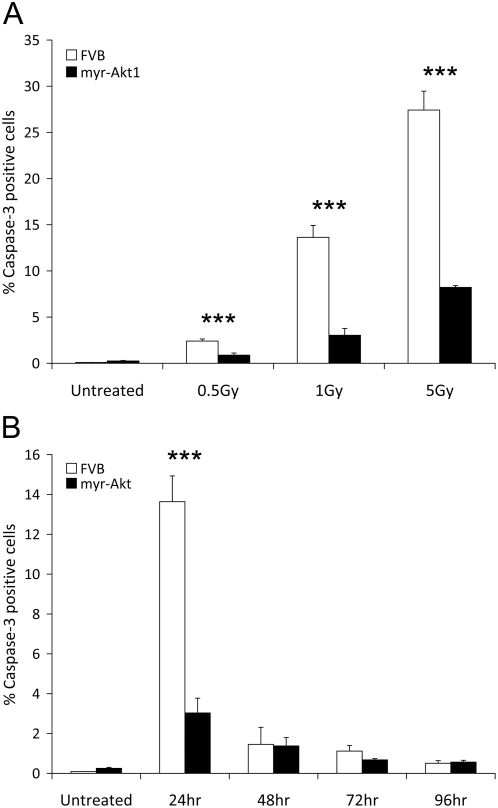
Reduced apoptosis in myr-Akt1 parotid glands following exposure to ionizing radiation. In A, four-week old female FVB and myr-Akt1 mice were exposed to 0.5, 1, or 5 Gy γ-radiation and parotid salivary glands were removed 24 hours post-irradiation. Tissues were embedded into paraffin and sections were stained for activated caspase-3. The number of caspase-3 positive cells is graphed as a percentage of the total number of cells per field of view. In B, four-week old female FVB and myr-Akt1 mice were exposed to 1 Gy γ-irradiation and parotid salivary glands were removed at 24, 48, 72 and 96 hours. Tissues were processed for activated caspase-3 immunohistochemistry as described in A. Five fields of view were quantitated for each tissue section and graphed using the averages and SEM from three mice per group. Significant differences (p≤0.05) were determined using a two sample t-test comparing FVB to myr-Akt1 and significant differences are marked with an asterisk (*).

We have previously described the phenotype of transgenic mice that express the myr-Akt1 transgene in either the mammary gland [16], or the salivary gland [14]. In this study, we have evaluated the extent of apoptosis in the parotid salivary gland following exposure of the head and neck region of control FVB and myr-Akt1 transgenic mice to increasing doses of γ-radiation. Analysis focused on the parotid salivary gland due to its high sensitivity to radiation and its functional importance in stimulated salivary flow. Apoptosis was quantified using immunohistochemical staining for activated caspase-3. Twenty-four hours after treatment, a significant (p≤0.05) level of apoptosis is detected in FVB control mice (Figure 1A) exposed to 0.5, 1 and 5 Gy doses of radiation that appears to be dose dependent. Approximately 2.4% of the parotid salivary cells are positive for activated caspase-3 following exposure to 0.5 Gy, 13% of the parotid salivary cells are positive for activated caspase-3 following exposure to 1 Gy and 27% are caspase-3 positive following exposure to 5 Gy γ-radiation (Figure 1A). Expression of myr-Akt1 significantly (p≤0.05) reduces the number of caspase-3 positive cells at all doses of radiation 24 hours after treatment; less than 1% of parotid salivary cells are apoptotic following exposure to 0.5 Gy, approximately 3% of parotid salivary cells are apoptotic after exposure to 1 Gy and approximately 8% of the parotid cells are positive for active caspase-3 following a single exposure to 5 Gy γ-irradiation (Figure 1A). Greater than 95% of the caspase-3 positive cells in the parotid gland are salivary acinar cells with few apoptotic ductal cells.

Because therapeutic doses administered to patients are kept below 2 Gy per exposure, we decided to evaluate apoptosis in the parotid gland and salivary function following a single low dose of radiation (1 Gy). The number of apoptotic cells was quantitated at 24 hour intervals following head and neck irradiation. The highest level of apoptosis in the irradiated parotid glands is observed at 24 hours post treatment and decreasing numbers of apoptotic cells are detected at the subsequent times examined (Figure 1B). Akt significantly (p≤0.05) suppresses the early induction of apoptosis (24 hr) when compared to FVB mice at the same time point. The level of apoptosis returns to untreated levels by 48–96 hours and no significant (p≤0.05) differences are observed between FVB and myr-Akt1 mice. This suggests that Akt diminishes the overall cell death in the tissue rather than delaying cell death. These data are consistent with numerous studies that have demonstrated that expression of a constitutively active mutant of Akt is able to suppresses apoptosis induced by many different stimuli both *in vitro* and *in vivo* [9]–[11].

### Preservation of salivary flow rates 3 days after γ-radiation exposure in myr-Akt1 mice

**Figure 2:**
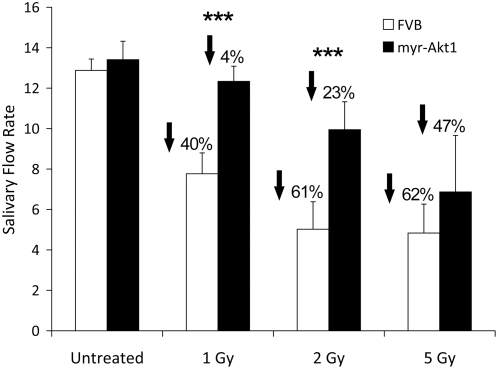
Preservation of salivary flow rates 3 days after single γ-radiation exposure in myr-Akt1 mice. Four-week old female FVB and myr-Akt1 mice were exposed to 1, 2, or 5 Gy γ-radiation. Three days after irradiation total saliva was collected (over a five minute period) following carbachol injection. Statistical analysis was performed using Student's t-test in Microsoft Excel. Results shown are from ten mice per group in the 1 and 2 Gy doses and four mice per group in the 5 Gy dose. Graphs represent averages and SEM from all mice. Significant differences (p≤0.05) were determined using a two sample t-test comparing FVB to myr-Akt1 and significant differences are marked with an asterisk (*).

Radiation-induced suppression of salivary function in the rat has been previously reported to occur almost immediately after exposure, reach a minimum at three days, and remain decreased for 180 days [7]. The significant differences in apoptosis detected between myr-Akt1 transgenic mice and FVB control mice (Figure 1) stimulated us to ask whether there was a difference in salivary flow rate between these two strains of mice. A significant decrease (40–62%) in salivary flow is detected in FVB control mice at each dose of radiation examined (Figure 2). Interestingly similar to a previous report using p53 null mice [17], apoptosis in the FVB mice appears to be dose dependent; however the reductions in salivary function in response to radiation do not appear to be dose dependent. In myr-Akt1 transgenic mice, the decrease in salivary flow rate is less severe (4–47%) with a significant (p≤0.05) improvement in salivary output at the lower doses of radiation (1–2 Gy). These data clearly indicate that, contrary to previous conclusions by other investigators, the extent of apoptosis induced in the salivary glands by head and neck irradiation may be causally related to salivary gland dysfunction.

### IGF1 activates Akt *in vivo* and preserves salivary flow rates 3 days after γ-radiation exposure

**Figure 3:**
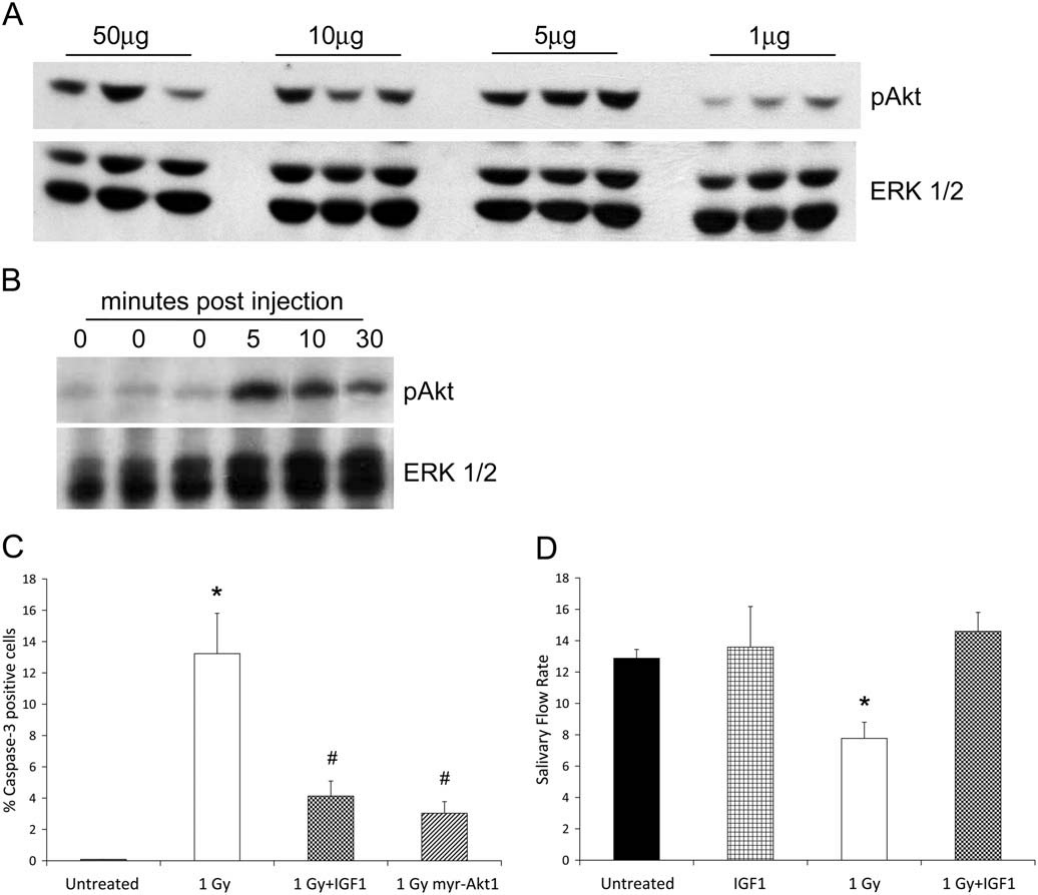
IGF1 activates Akt *in vivo* and preserves salivary flow rates 3 days after single γ-radiation exposure. In A, FVB mice received an injection of 1, 5, 10, or 50 µg recombinant IGF1. Tissue lysates were collected for immunoblotting five minutes after injection and membranes were probed for activation of Akt using a phosphorylation specific antibody. Results shown are representative of three independent experiments. In B, FVB mice received an injection of 5 µg recombinant IGF1 and tissue lysates were collected for immunoblotting 0, 5, 10 or 30 minutes after injection. Membranes were probed for activated Akt as described in A. Membranes were stripped and re-probed with a total antibody against ERK1/2 as a loading control in both A and B. Results shown are representative of three independent experiments. In C, four-week old female FVB mice were injected with 5 µg recombinant IGF1 immediately prior to treatment with 1 Gy γ-radiation. Salivary glands were removed 24 hours post-irradiation and stained for activated caspase-3 as described in Figure 1A. Graph represents averages and SEM from at least three mice/treatment. (*) indicates significant difference (p≤0.05) from untreated FVB and (#) indicates significance between 1 Gy FVB and 1 Gy IGF1 or 1 Gy myr-Akt1. In D, four-week old female mice were injected with 5 µg recombinant IGF1 and treated with radiation as described in C. Total saliva was collected following carbachol injection 3 days after radiation exposure as described in Figure 2. Statistical analysis was performed using Student's t-test in Microsoft Excel. Results shown are from ten irradiated FVB mice and eight IGF1 plus irradiation mice and graphed using the averages and SEM from all mice. Significant differences (p≤0.05) were determined using a two sample t-test comparing FVB to myr-Akt1 and significant differences are marked with an asterisk (*).

We have previously shown that IGF1 induces activation of Akt in salivary acinar cells and suppresses apoptosis induced by DNA damage (etoposide) *in vitro* [12]. Stimulation of cells with both IGF1 and EGF produced a synergistic activation of Akt and suppression of apoptosis [12]. We therefore determined whether intravenous injections of recombinant IGF1 into the tail vein of mice would activate endogenous Akt in the parotid salivary gland similar to a previous study [18]. FVB mice were injected with 1, 5, 10, or 50 µg recombinant IGF1 and the activation of Akt was examined in the parotid gland five minutes after injection. Injection of 5 µg IGF1 results in the maximal activation of Akt as determined by immunoblotting with anti-phospho-Akt (threonine^473^) antibody (Figure 3A).

We also determined the kinetics with which Akt was activated following injection of FVB mice with 5 µg IGF1. Parotid glands were removed at 5, 10, and 30 minutes post injection, tissue lysates prepared, and the activation of Akt examined by immunoblotting with anti-phospho-Akt (threonine^473^) antibody (Figure 3B). Maximal activation of Akt in the parotid gland is detected five minutes after injection of IGF1, and the amount of phosphorylated Akt declines after this time. However, phosphorylated Akt could still be detected thirty minutes following administration of IGF1, and may remain activated for up to four hours post-injection (data not shown).

To determine whether acute administration of mice with IGF1 could suppress radiation-induced salivary gland hypofunction, FVB mice were anesthetized with avertin, injected with 5 µg IGF1, and immediately exposed to 1 Gy radiation. Parotid salivary glands were removed 24 hours later to quantitate the level of apoptosis using immunohistochemistry for activated caspase-3 (Figure 3C). Approximately 4% of the parotid salivary cells are apoptotic in mice receiving IGF1 prior to irradiation which is significantly (p≤0.05) lower than the 13% observed in irradiated FVB. The level of radiation-induced apoptosis in the IGF1 injected is not significantly different than the myr-Akt1 mice (3%).

We also evaluated salivary flow rates three days after exposure to γ-radiation. Injection of mice with 5 µg IGF1 alone had no effect upon the salivary flow rate three days following injection of the mice (Figure 3D). Following radiation, there is a 40% decrease in the salivary flow rate compared to unirradiated control mice (Figure 3D), which is statistically different (p≤0.05). Injection of mice with a single dose of 5 µg recombinant IGF1 completely suppresses the radiation-induced reduction in salivary flow rate at this early time point (Figure 3D).

### Preservation of salivary flow rates 30 days after γ-radiation exposure by myr-Akt1 or injection with IGF1

**Figure 4:**
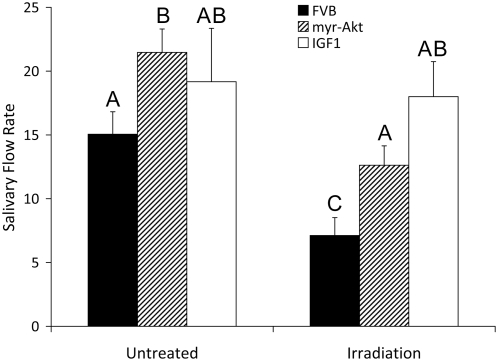
Preservation of salivary flow rates 30 days after single γ-radiation exposure by myr-Akt1 or injection with IGF1. Four-week old female FVB, myr-Akt1 and FVB mice injected with 5 µg recombinant IGF1 were exposed to 1 Gy γ-radiation. IGF1 injections were performed immediately prior to radiation exposure as described in Figure 3. Thirty days after exposure to γ-radiation total saliva was collected following carbachol injection as described in Figure 2. Statistical analysis was performed using multiple comparison testing in the SAS system. Results shown are from ten irradiated FVB mice, ten irradiated myr-Akt1 mice and eight IGF1 plus irradiation mice and graphed using the averages and SEM from all mice. Treatment groups with the same letters are not significantly different from each other.

One hallmark of radiation-induced salivary gland dysfunction is that the effect is chronic and there is no recovery in the production of saliva over time [7]. Therefore we re-evaluated salivary flow rates in irradiated FVB, myr-Akt1 transgenic and IGF1 pretreated mice 30 days post γ-radiation to determine if the trends observed three days following irradiation would be retained over time (Figure 4). There was a slight increase in salivary flow rates in all unirradiated at day 30 when compared to the flow rates determined in unirradiated mice on day 3. The flow rate in the unirradiated myr-Akt1 transgenic mice is higher than the unirradiated FVB control mice at the thirty day time point, and this difference is significant (Treatment groups with the same letters are not significantly different from each other). There is no statistical difference in the salivary flow rate between unirradiated FVB control mice and mice injected with IGF1 at the thirty day time point. There is a 60% decrease in the salivary flow rate in irradiated FVB mice compared to control mice at the thirty day time point. In irradiated myr-Akt1 mice, there is a 16% decrease in salivary flow when compared to FVB untreated controls that is not significantly different; however it is significantly lower than unirradiated myr-Akt1. Remarkably, FVB mice injected with a single dose of IGF1 prior to γ-radiation demonstrate no decrease in salivary flow rate 30 days after irradiation compared to control mice (FVB or IGF1 injected).

**Figure 5:**
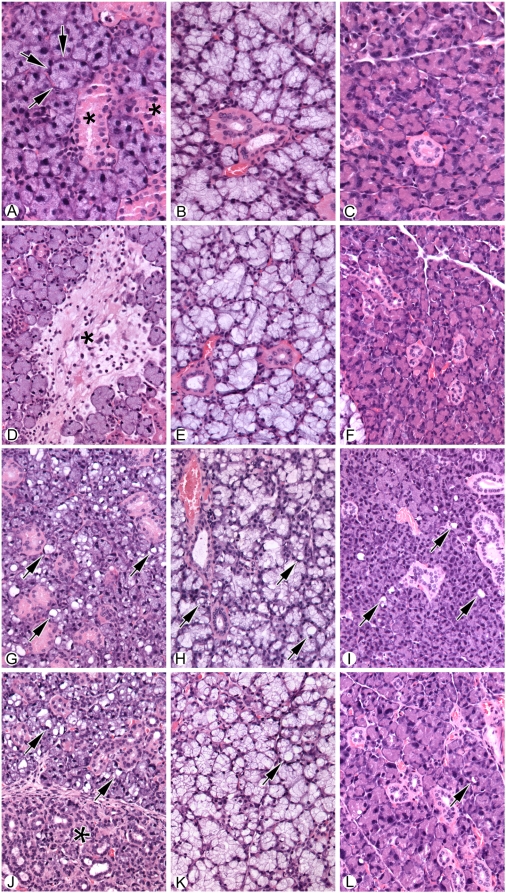
Histological analysis of salivary glands from normal and irradiated salivary glands from untreated and IGF1-treated mice. A) Normal submandibular salivary gland showing basic structure of acini (arrows) and ducts (asterisk). B) Normal sublingual salivary gland. C) Normal parotid gland. D) Submandibular salivary gland thirty days following exposure to 5 Gy radiation. Note area of focal fibrosis and associated inflammatory cells (asterisk). E) Sublingual salivary gland after thirty days following exposure to 5 Gy radiation. No significant morphological change is seen. F) Parotid salivary gland after exposure to radiation. No Significant morphological change is seen. G) Submandibular gland thirty days following injection with 5 ug IGF1. Note the prominent vacuolization of the glandular acini (Arrows). H) Sublingual gland thirty days following injection with 5 ug IGF1 introduction. Note the prominent vacuolization of the glandular acini (Arrows). I) Parotid gland thirty days following injection with 5 ug IGF1. Note the mildly increased vacuolization of the glandular acini, although less than that observed in either the submandibular or sublingual salivary glands (Arrows). J) Submandibular gland thirty days following injection with 5 ug IGF1 immediately prior exposure to 5 Gy radiation. The left part of the photograph (*) shows atrophy of the acini with mild chronic inflammation. The right side of the picture shows increased vacuolization in the viable acini (arrows). K) Sublingual gland thirty days following injection with 5 ug IGF1 immediately prior to exposure to 5 Gy radiation. Increased vacuolization is noted, however, no significant atrophy is seen. L) Parotid gland thirty days following injection with 5 ug IGF1 immediately prior to exposure to 5 Gy radiation. Note the mildly increased vacuolization with no significant atrophy. For Panels A–C, the magnification is 40×, while the magnification D–L is 20×.

Histological analysis of the salivary glands from the different groups of mice thirty days post treatment (control, irradiated, IGF-1 treated but unirradiated, and IGF-1 treated and irradiated) were conducted (Figure 5). Consistent with previous observations in irradiated rats [19] only minimal or mild focal fibrosis was observed in the submandibular glands of irradiated mice; a rare large area of fibrosis, which is not typical of what is generally observed, is shown in Figure 5D. The extent of fibrosis did not appear to be significantly different between mice pretreated with IGF-1 prior to irradiation (Figure 5D versus 5J). Radiation-induced fibrosis was not apparent in either the sublingual or parotid glands (compare 5B versus 5E and 5C versus 5F). Pretreatment of mice with IGF-1 did result in some increase in the large vacuoles in acinar cells in each of the salivary glands (Figure 5G, H, and I). These vacuoles are not prominent in the submandibular gland, although they are present in each type of salivary gland examined (Figure 5G–I). We do not understand the mechanism that underlies the ability of IGF1 to induce the appearance of these vacuoles in salivary acinar cells; however, they have no effect upon production of saliva in the unirradiated mice (Figure 4D and E).

## Discussion

These data indicate that irradiation of FVB mice results in a significant induction of apoptosis and salivary gland dysfunction characterized by a decreased salivary flow rate that persists for at least 30 days. Expression of activated myr-Akt1, or pretreatment of mice with recombinant IGF1, which activates Akt, significantly decreases the number of apoptotic cells, and significantly suppresses radiation-induced salivary gland dysfunction. We note that there is a significant difference in the extent of protection offered by expression of activated Akt versus that afforded by injection of IGF1 with the latter providing significantly greater protection. IGF1 is able to activate a broader spectrum of signaling pathways, including the MAP kinase pathway, and its greater protection may reflect its ability to both suppress apoptosis as well as induce proliferation of cells. We suggest that this difference may account for the greater protection from irradiation-induced salivary hypofunction offered by IGF1. Furthermore, our results indicate that means to suppress apoptosis in salivary epithelial cells could prevent inadvertent salivary gland dysfunction occurring secondary to head and neck irradiation.

In this study we have focused primarily upon the parotid gland. The submandibular glands contribute approximately two-thirds of unstimulated saliva volume, whereas parotid glands contribute the majority of stimulated saliva volume[20], [21]. Furthermore Humphries et al have shown that the extent of irradiation induced apoptosis is ten-fold lower in the submandibular gland compared to the parotid gland [22]. For these reason we feel that focusing our analysis upon the parotid gland is justified.

In recent years, a significant amount of attention has been focused upon understanding the molecular changes underlying the adverse effects of therapeutic radiation which negatively impact a patient's quality of life as a means for developing useful therapeutic interventions. Recent studies have suggested that gastrointestinal syndrome induced by whole body irradiation may result from radiation-induced apoptosis of endothelial cells [23]. This observation is contrary to previous hypotheses that gastrointestinal syndrome was caused by the failure of stem cells within the intestinal crypt to proliferate and reconstitute the crypt-villus unit. The ability of a peptide agonist to Toll-like receptor 5 (CBLB502) to preserving endothelial function in the gastrointestinal tract and prevent gastrointestinal syndrome has provided confirmatory evidence that endothelial cells likely represent one important target underlying gastrointestinal syndrome resulting from whole body irradiation [24]. Although this study reported that CBLB502 did not affect tumor clearance [24], it is important to note that tumor response to radiation is also influenced by endothelial apoptosis [25]. These results indicate the clinical importance of preventing radiation-induced death of off-target cells in the treatment of cancer patients.

The situation in the salivary gland is somewhat analogous in that salivary gland hypofunction could result from loss of tissue stem cells that are capable of repopulating the damaged cells, damage to secretory acinar epithelial cells, loss of endothelial cells, or loss of nerve cells required for induction of secretion. Of these four possibilities, it does not appear that radiation induces changes to the blood supply [26], [27], or the innervation of the salivary gland [28], although it is possible that a reexamination of these points using recently generated reagents or techniques could alter this conclusion. Our results suggest that apoptosis of epithelial cells may be important in radiation-induced salivary gland hypofunction and the resulting diminished quality of life for head and neck cancer patients. Our results also indicate that IGF1 can be used to prevent salivary epithelial cell apoptosis and loss of glandular function, which represents a significant difference from studies on the role of endothelial cell apoptosis in gastrointestinal syndrome.

The tumor suppressor gene p53 has also been shown to be involved in the radiosensitivity of many tissues [29], and loss of p53 function represents an important step in tumor progression. Transgenic mice expressing an inducible form of p53 have been used to evaluate radiation-induced lymphoma and these studies concluded that delaying p53 expression until after the apoptotic response had no effect on eventual tumors caused by radiation damage [30]. These results indicate that the role of p53 in apoptosis and tumor suppression are separate functions of p53 [30]. We have previously demonstrated that MDM2 is a critical target of Akt in the suppression of DNA damage-induced apoptosis in salivary acinar cells [14]. We observed the Akt-dependent phosphorylation of MDM2 in the salivary glands of myr-Akt1 transgenic mice which resulted in complete absence of p53 from these glands, as well as a significant decrease in two other p53 family members, p63 and p73[14]. The loss of p53 also resulted in the decreased expression of p53 target genes such a p21^WAF1^ [14]. These results suggest that the p53 pathway is critical in regulating the radiosensitivity of the salivary glands[14]. Recently it has been reported that expression of p53 is required for the acute and chronic loss of salivary function following irradiation [17]. Temporary suppression of radiation-induced apoptosis of salivary acinar cells could prevent inadvertent salivary gland dysfunction occurring secondary to head and neck irradiation without predisposing the salivary glands to eventual tumorigenesis.

Clearly our work highlights a first step in an effort to improve the treatment of head and neck cancer patients by preventing the initial loss of salivary function. Identification of the pathways mediating the IGF1-Akt preservation of salivary function will allow specific targeting to the salivary glands without modifying tumor therapies. Current efforts are directed towards building a preclinical model to evaluate the effectiveness of this approach.

## Methods

### Mice

Myr-Akt1 transgenic mice were generated using standard techniques as described previously by the Transgenic Mouse Core of the University of Colorado Cancer Center [16]. Genomic tail DNA was extracted and transgenic mice were identified using PCR as previously described [16]. Non-transgenic littermates were used as controls in all experiments. Animals were maintained and treated in accordance with protocols approved by the University of Colorado Health Sciences Center Laboratory Animal Care and Use Committee.

### γ-radiation treatment

For in vivo experiments, four-week old female FVB and myr-Akt1 transgenic mice were anesthetized with avertin (0.4–0.6 mg/gm, IP) and the head and neck region was exposed to ionizing radiation using a RS 2000 Biological Irradiator [22]. The rest of the body was shielded with ∼6 mm lead foil and sheets to avoid systemic effects of γ-irradiation. Mice completely shielded with lead exhibited no adverse effects of radiation suggesting that the shielding used was sufficient to prevent systemic damage. For IGF1 injections (GroPrep, Adelaide, Australia), mice were anesthetized with avertin and tails were warmed in water to allow dilation of blood vessels. Mice were injected with a total volume of 100 µl of IGF1 diluted in sterile PBS and immediately placed into the radiation chamber.

### Histology

Submandibular, sublingual, and parotid salivary gland tissues were fixed in 10% neutral buffered formalin, and then embedded in paraffin. Tissue sections were cut at 4 µm and processed for standard staining with hematoxylin and eosin by the Histology Service of the Department of Pathology at the University of Colorado School of Medicine. Tissue sections were observed by standard light microscopy and photomicrographs were taken with an Olympus BX51 microscope with Spot Diagnostic imaging software.

### Activated caspase-3 staining

Salivary glands were removed at various time points post-irradiation, fixed in 10% neutral buffered formalin, and then embedded in paraffin. Slides were stained for activated caspase-3 (Cell Signaling #9661; Beverly, MA) as previously described [14], [22]. Cell counts were performed on parotid gland sections from a minimum of five fields of view per slide from three mice per treatment (total cells counted ranged from 1800–2500 per mouse).

### Saliva collection

Mice were injected intraperitoneally with 0.25 mg carbachol per kg body weight [31]. Saliva was collected immediately following injection for five min and chilled on ice as previously described [14].

### Statistics

Statistical evaluation for multiple comparisons (Figure 4) was performed in SAS using general linear means and the least squares means for effect to control for Type I errors. All other statistical calculations for Figures 1, 2 and 3 were performed using a two sample t-test in Microsoft Excel to compare FVB vs. myr-Akt1 or IGF1.
